# Supplementary material for: EAG Responses of Adult Lobesia botrana Males and Females Collected from Vitis vinifera and Daphne gnidium to Larval Host-Plant Volatiles and Sex Pheromone
Source: Insects. 2019 Sep 2;10(9):281. doi: 10.3390/insects10090281 (PMC6780690; doi:10.3390/insects10090281)

EAG responses of adult *Lobesia botrana* males and females collected from *Vitis vinifera* and *Daphne gnidium* to larval host-plant volatiles and sex pheromone

Alicia Pérez-Aparicio 1, Luis M. Torres-Vila, and César Gemeno

Supplementary material

Table S1. Pairwise significance tests for the factors A) larval host-plant, B) odorant by sex, and C) sex by odorant in the ANOVA performed with the complete dataset (Table 3A, main text). Different letters in each section indicate significant differences among groups (Tukey´s test, P < 0.05)

**A.** Larval host-plant						
**host**	**response**	**SE**	**df**	**lower.CL**	**upper.CL**	**.group**
Vitis	0.400	0.0204	239	0.361	0.442	a
Daphne	0.302	0.0154	239	0.273	0.334	b
**B.** Odorant by sex						
**sex = female**						
**odor**	**response**	**SE**	**df**	**lower.CL**	**upper.CL**	**.group**
far	0.781	0.1447	239	0.5418	1.125	a
msl	0.571	0.1058	239	0.3961	0.822	ab
1ol	0.499	0.0926	239	0.3466	0.720	ab
lnl	0.426	0.0789	239	0.2953	0.613	ab
ben	0.380	0.0705	239	0.2637	0.548	ab
dmn	0.330	0.0611	239	0.2288	0.475	ab
2eh	0.301	0.0559	239	0.2092	0.434	bc
lox	0.273	0.0507	239	0.1898	0.394	bc
ebz	0.272	0.0505	239	0.1889	0.392	bc
major	0.138	0.0256	239	0.0957	0.199	cd
minac	0.110	0.0205	239	0.0766	0.159	d
minoh	0.107	0.0198	239	0.0740	0.154	d
**sex = male**						
**odor**	**emmean**	**SE**	**df**	**lower. CL**	**upper. CL**	**group**
major	3.629	0.6141	239	25.999	5.065	a
minac	0.832	0.1408	239	0.5960	1.161	b
far	0.807	0.1366	239	0.5784	1.127	bc
1ol	0.392	0.0664	239	0.2809	0.547	bcd
lnl	0.367	0.0621	239	0.2631	0.512	cd
ben	0.323	0.0547	239	0.2317	0.451	d
msl	0.289	0.0490	239	0.2074	0.404	d
lox	0.268	0.0454	239	0.1921	0.374	d
minoh	0.268	0.0453	239	0.1917	0.373	d
dmn	0.261	0.0441	239	0.1867	0.364	d
ebz	0.195	0.0330	239	0.1398	0.272	d
2eh	0.184	0.0311	239	0.1318	0.257	d
**C.** Sex by odorant						
**odor = 1ol**						
**sex**	**response**	**SE**	**df**	**lower.CL**	**upper.CL**	**.group**
f	0.499	0.0926	239	0.3466	0.720	a
m	0.392	0.0664	239	0.2809	0.547	a
**odor = 2eh**						
**sex**	**response**	**SE**	**df**	**lower.CL**	**upper.CL**	**.group**
f	0.301	0.0559	239	0.2092	0.434	a
m	0.184	0.0311	239	0.1318	0.257	a
**odor = ben**						

**sex**	**response**	**SE**	**df**	**lower.CL**	**upper.CL**	**.group**
f	0.380	0.0705	239	0.2637	0.548	a
m	0.323	0.0547	239	0.2317	0.451	a

odor = dmn

**sex**	**response**	**SE**	**df**	**lower.CL**	**upper.CL**	**.group**
f	0.330	0.0611	239	0.2288	0.475	a
m	0.261	0.0441	239	0.1867	0.364	a

odor = ebz

**sex**	**response**	**SE**	**df**	**lower.CL**	**upper.CL**	**.group**
f	0.272	0.0505	239	0.1889	0.392	a
m	0.195	0.0330	239	0.1398	0.272	a

odor = far

**sex**	**response**	**SE**	**df**	**lower.CL**	**upper.CL**	**.group**
f	0.807	0.1366	239	0.5784	1.127	a
m	0.781	0.1447	239	0.5418	1.125	a

odor = lnl

**sex**	**response**	**SE**	**df**	**lower.CL**	**upper.CL**	**.group**
f	0.426	0.0789	239	0.2953	0.613	a
m	0.367	0.0621	239	0.2631	0.512	a

odor = lox

**sex**	**response**	**SE**	**df**	**lower.CL**	**upper.CL**	**.group**
f	0.273	0.0507	239	0.1898	0.394	a
m	0.268	0.0454	239	0.1921	0.374	a

odor = major

**sex**	**response**	**SE**	**df**	**lower.CL**	**upper.CL**	**.group**
f	3.629	0.6141	239	25.999	5.065	a
m	0.138	0.0256	239	0.0957	0.199	b

odor = minac

**sex**	**response**	**SE**	**df**	**lower.CL**	**upper.CL**	**.group**
f	0.832	0.1408	239	0.5960	1.161	a
m	0.110	0.0205	239	0.0766	0.159	b

odor = minoh

**sex**	**response**	**SE**	**df**	**lower.CL**	**upper.CL**	**.group**
f	0.268	0.0453	239	0.1917	0.373	a
m	0.107	0.0198	239	0.0740	0.154	b

odor = msl

**sex**	**response**	**SE**	**df**	**lower.CL**	**upper.CL**	**.group**
f	0.571	0.1058	239	0.3961	0.822	a
m	0.289	0.0490	239	0.2074	0.404	b

Table S2. Pairwise significance tests for the factors A) larval host-plant, B) plant odorant, and C) sex in the ANOVA performed with the *plant dataset* (Table 3B, main text). Different letters in each section indicate significant differences among groups (Tukey´s test, P < 0.05)

**A.** Larval host-plant						
**host**	**response**	**SE**	**df**	**lower.CL**	**upper.CL**	**.group**
vitis	0.416	0.0261	187	0.368	0.471	a
daphne	0.300	0.0188	187	0.265	0.339	b
**B.** Plant odorant						
**odor**	**response**	**SE**	**df**	**lower.CL**	**upper.CL**	**.group**
far	0.804	0.1067	187	0.619	1.045	a
1ol	0.443	0.0587	187	0.341	0.575	b
msl	0.399	0.0529	187	0.307	0.518	bc
lnl	0.397	0.0527	187	0.306	0.516	bc
ben	0.352	0.0467	187	0.271	0.457	bc
dmn	0.293	0.0389	187	0.226	0.381	bc
lox	0.274	0.0363	187	0.211	0.355	bc
2eh	0.233	0.0309	187	0.179	0.303	c
ebz	0.230	0.0305	187	0.177	0.298	c
**C.** Sex						
**sex**	**response**	**SE**	**df**	**lower.CL**	**upper.CL**	**.group**
f	0.401	0.0263	187	0.352	0.456	a
m	0.311	0.0186	187	0.276	0.350	b

Table S3. Pairwise significance tests for the pheromone compound*sex interaction [A) Pheromone compound by sex, B) sex compound by pheromone] in the ANOVA performed with the *pheromone dataset* (Table 3C, main text). Different letters in each section indicate significant differences among groups (Tukey´s test, P < 0.05)

**A.** Pheromone compound by sex						
**sex = female**						
**odor**	**response**	**SE**	**df**	**lower.CL**	**upper.CL**	**.group**
major	0.138	0.0194	60	0.1040	0.183	a
minac	0.110	0.0155	60	0.0833	0.146	a
minoh	0.107	0.0150	60	0.0805	0.141	a
**sex = male**						
**odor**	**response**	**SE**	**df**	**lower.CL**	**upper.CL**	**.group**
major	3.629	0.4665	60	28.059	4.693	a
minac	0.832	0.1069	60	0.6432	1.076	b
minoh	0.268	0.0344	60	0.2069	0.346	c
**B.** Sex compound by pheromone						
**Major pheromone compound (*E*7, *Z*9-12:Ac)**						
**sex**	**response**	**SE**	**df**	**lower.CL**	**upper.CL**	**.group**
m	3.629	0.4665	60	28.059	4.693	a
f	0.138	0.0194	60	0.1040	0.183	b
**Minor pheromone compound *Z*9-12:Ac**						
**sex**	**response**	**SE**	**df**	**lower.CL**	**upper.CL**	**.group**
m	0.832	0.1069	60	0.6432	1.076	a
f	0.110	0.0155	60	0.0833	0.146	b
**Minor pheromone compound (*E*7, *Z*9-12:OH)**						
**sex**	**response**	**SE**	**df**	**lower.CL**	**upper.CL**	**.group**
m	0.268	0.0344	60	0.2069	0.346	a
f	0.107	0.0150	60	0.0805	0.141	b

Figure S1. EAG trace of a *Lobesia botrana* male collected from *Vitis vinifera* (VG2 population, EAG file **130601407**). Stimuli: a) (*E*)-β-farnesene, b) major sex pheromone compound (*E*7,*Z*9-12:Ac), c) 1-octen-3-ol, d) puff and solvent control (*n*-hexane), e) methyl salicylate, f) linalool, g) minor pheromone compound (*E*7,*Z*9-12:OH), h) benzothiazole, i) 2-ethylhexan-1-ol, j) (*E*)-4,8-dimethyl-1,3,7-nonatriene. Top trace, EAG; bottom trace, puffs (0.5 s duration).

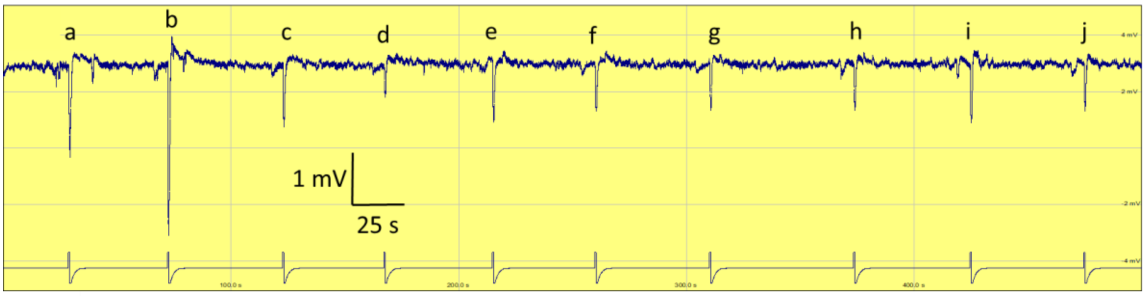

Figure S2. Electroantennogram responses of *L. botrana* males and females collected in *Vitis vinifera* or *Daphne gnidium* to host-plant odorants and individual sex pheromone compounds. Plant compounds were *V. vinifera* specific [1-octen-3-ol, (*E*)-β-farnesene, (*E*)-4,8-dimethyl-1,3,7-nonatriene], *D. gnidium*-specific (2-ethyl-hexan-1-ol, benzothiazole, linalool-oxide, ethyl benzanoate) or common to both (linalool and methyl salicylate).

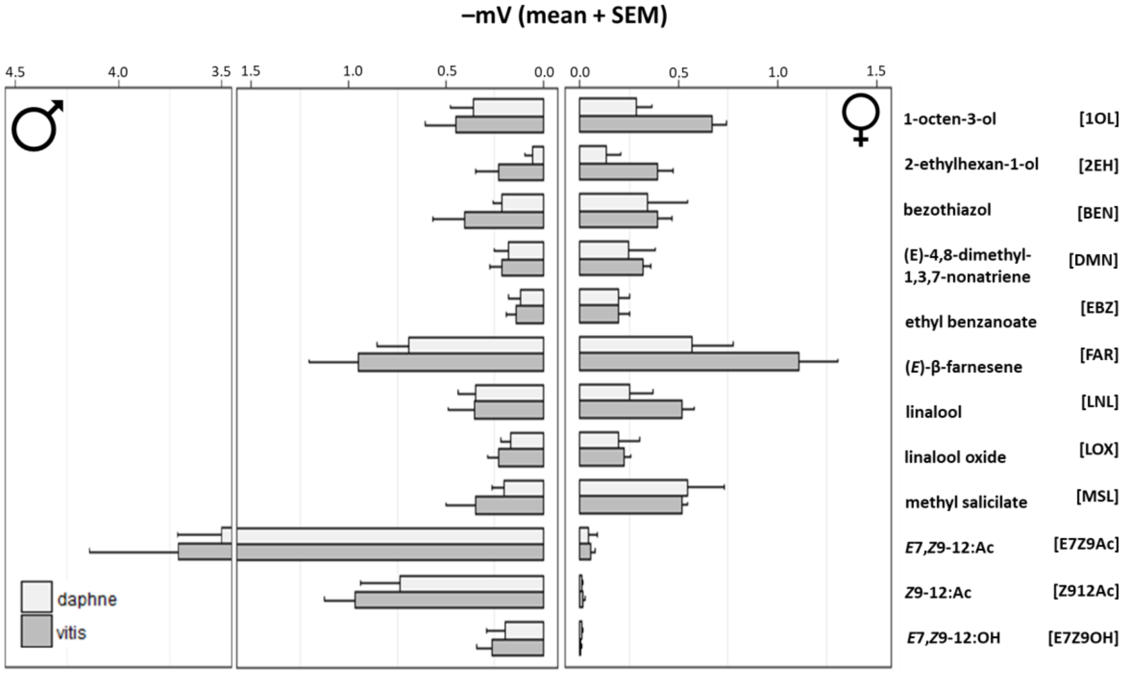

Supplement: Supplementary file 1 [file insects-10-00281-s001.pdf]
